# Chronic disease, risk factors and disability in adults aged 50 and above living with and without HIV: findings from the Wellbeing of Older People Study in Uganda

**DOI:** 10.3402/gha.v9.31098

**Published:** 2016-05-24

**Authors:** Joseph O. Mugisha, Enid J. Schatz, Madeleine Randell, Monica Kuteesa, Paul Kowal, Joel Negin, Janet Seeley

**Affiliations:** 1 MRC/UVRI, Uganda Research Unit on AIDS, Uganda; 2 Department of Health Sciences, University of Missouri Columbia, Missouri, USA; 3 School of Public Health, University of Sydney, Australia; 4 World Health Organization, Study on global AGEing and adult health, Geneva, Switzerland; 5 Research Centre for Gender, Health and Ageing, University of Newcastle, Australia; 6 London School of Hygiene and Tropical Medicine, London UK

**Keywords:** Africa, aging, aging disability, HIV/AIDS, older adults, non-communicable diseases, Uganda

## Abstract

**Background:**

Data on the prevalence of chronic conditions, their risk factors, and their associations with disability in older people living with and without HIV are scarce in sub-Saharan Africa.

**Objectives:**

In older people living with and without HIV in sub-Saharan Africa: 1) to describe the prevalence of chronic conditions and their risk factors and 2) to draw attention to associations between chronic conditions and disability.

**Methods:**

Cross-sectional individual-level survey data from people aged 50 years and over living with and without HIV were analyzed from three study sites in Uganda. Diagnoses of chronic conditions were made through self-report, and disability was determined using the WHO Disability Assessment Schedule (WHODAS). We used ordered logistic regression and calculated predicted probabilities to show differences in the prevalence of multiple chronic conditions across HIV status, age groups, and locality. We used linear regression to determine associations between chronic conditions and the WHODAS.

**Results:**

In total, 471 participants were surveyed; about half the respondents were living with HIV. The prevalence of chronic obstructive pulmonary disease and eye problems (except for those aged 60–69 years) was higher in the HIV-positive participants and increased with age. The prevalence of diabetes and angina was higher in HIV-negative participants. The odds of having one or more compared with no chronic conditions were higher in women (OR 1.6, 95% CI 1.1–2.3) and in those aged 70 years and above (OR 2.1, 95% CI 1.2–3.6). Sleep problems (coefficient 14.2, 95% CI 7.3–21.0) and depression (coefficient 9.4, 95% CI 1.2–17.0) were strongly associated with higher disability scores.

**Conclusion:**

Chronic conditions are common in older adults and affect their functioning. Many of these conditions are not currently addressed by health services in Uganda. There is a need to revise health care policy and practice in Uganda to consider the health needs of older people, particularly as the numbers of people living into older age with HIV and other chronic conditions are increasing.

## Introduction

Chronic diseases are illnesses or conditions that require ongoing medical attention and affect a person's daily life (1). Chronic diseases include cancers, cardiovascular diseases, chronic respiratory diseases, diabetes, hypertension, mental disorders, and stroke. Other chronic impairments that commonly affect people include arthritis; rheumatism; and dental, vision, stomach, and intestinal problems (2). In African countries, improved access to antiretroviral treatment (ART) is increasing survival for those with the human immunodeficiency virus (HIV). Consequently, HIV is now considered a chronic condition in many settings (3).

With shifts in the global burden of disease, chronic diseases represent a substantial proportion of illnesses even in low- and middle-income countries (LMICs) (4). Few studies, however, have used individual-level data to elucidate the prevalence of chronic conditions, risk factors, and disability associated with chronic diseases in older people in LMICs, and such research is particularly scarce in sub-Saharan Africa. Comprehensive studies on chronic diseases in LMICs primarily have concentrated on younger and middle-aged people (5–10) with relatively few focusing on older adults (2, 9, 11, 12).

In sub-Saharan Africa, the number and proportion of older people is increasing and is projected to continue to grow in coming decades (13, 14). This makes it particularly important to understand how chronic disease impacts on older Africans’ lives. As African populations age, the prevalence of individuals with chronic conditions in these settings is likely to increase. In Uganda, for example, the population of older people has continued to grow rapidly (15). In addition, the number of older people living with HIV in Uganda is also increasing (16) in line with a global trend (17–19).

A number of studies have been conducted in sub-Saharan Africa on chronic conditions in adults (7–9, 20–25). However, few provide information on concurrent chronic conditions, including HIV (23), and fewer still have simultaneously examined chronic diseases in older people living with and without HIV (26). In Uganda, as well, there are few data on health differences in chronic conditions between older persons living with and without HIV (27–29).

Chronic diseases can affect people of all age groups, but they are more common and more likely to have negative consequences in older adults. A 2005 study of mortality and the burden of disease predicted an increase in deaths for all ages worldwide due to chronic diseases (excluding HIV) from 35 million deaths in 2005 to 41 million deaths in 2015 (30). Nearly 60% of the deaths in each year are estimated to occur among those aged 70-plus. Research from southern Africa shows that chronic diseases (not including HIV) are more prevalent among those aged 50-plus compared to those aged 18–49 (12). Another study in South Africa showed that there were more chronic conditions (excluding HIV) in later older age (65-plus) than early older age (ages 50–65) (9).

With the exception of HIV, many chronic diseases share common risk factors. These include excessive alcohol use, tobacco use, unhealthy diets, and physical inactivity (31). Current health behaviors, as well as the accumulated impact of a lifetime of harmful health behaviors, contribute to the higher likelihood of contracting a chronic condition in older age (32, 33). Because the majority of these risk factors are related to individual health behaviors, most are potentially amenable to behavioral interventions (34).

Using a unique dataset from Uganda, this paper describes the prevalence of chronic diseases, including angina; arthritis; chronic obstructive pulmonary disease (COPD); depression; diabetes mellitus; and hypertension, stroke, and vision problems, in older people living with and without HIV. We also describe the prevalence of related risk factors and association between chronic disease and disability, using the World Health Organization Disability Assessment Schedule (WHODAS 2.0) to measure disability (35). This paper adds to the limited body of literature on the prevalence and risk factors of chronic conditions and how these impact on disability in older Africans living with and without HIV.

## Methods

Data for this analysis came from the second wave of the longitudinal World Health Organization Study on global AGEing and adult health (SAGE)-Wellbeing of Older People Study (WOPS). The SAGE-WOPS HIV study in Uganda was implemented in people aged 50 plus. To date, two waves of data are available: the first wave (WOPS1) conducted in 2009–2010 and the second wave (WOPS2) conducted in 2012–2013. Details of the initial WOPS recruitment are described elsewhere (26). Although data from two waves of WOPS are available, only data from WOPS2 are analyzed here because of inconsistencies in available variables across the two waves. We therefore present findings on a fuller set of more recent variables rather than longitudinal data on a limited set of variables.

Interviews were conducted in three sites on the shores of Lake Victoria – in the Kalungu and Masaka districts and another in the Wakiso District, near Entebbe. The study setting, study population, and data collection are also described elsewhere (26, 36). Briefly, the WOPS1 sample consisted of 510 older people (61.2% female, mean age 65 and age range 50–96 years). These included 1) older persons who were living with HIV but not yet on ART; 2) older persons living with HIV and on ART for at least 1 year; 3) older persons who had a child living with HIV; 4) older persons who had a child who died of AIDS-related illness; and 5) older persons who were not HIV-positive themselves but had not lost a child due to HIV infection.

During WOPS2, we re-interviewed those respondents who were still living in the area; 148 respondents were lost to follow-up (these included 67 who had died, 25 who emigrated from the study area, 17 who were found but refused to participate, 9 who were too sick to participate, 4 who had travelled on the day of the interviews, 4 who were too busy to participate in the interviews, and 22 who could not be located). The follow-up rate was over 70%. In WOPS2, we recruited an additional 100 older people living with HIV attending the AIDS Support Organization (TASO), a non-governmental organization (NGO) in Masaka town, close to the Kalungu District site. All the new recruits were randomly selected from older people attending TASO. These additional recruits increased the number of people living with HIV in the cohort. In order to avoid misclassification of the study groups, all older people who were HIV negative in WOPS1 were retested for HIV using the Uganda Ministry of Health algorithms for rapid HIV testing (37). The sample in this study is stratified by HIV status between all those who were living with HIV either in WOPS1 or WOPS2, and those who were HIV negative in WOPS1 and remained so at the time of testing in WOPS2.

### Data collection

Study participants were either interviewed from home or from a central hub (a central location in their village), where a house was rented for survey activities. The interviews were conducted by trained interviewers using a validated questionnaire. After conducting the interviews, the interviewers measured weight, height, blood pressure, grip strengths, walking speed, and conducted a visual acuity test. The WOPS questionnaire and other data collection instruments were adapted from the WHO SAGE (38). All instruments were pretested and piloted prior to use (26).

### Variables

The components of the study questionnaire analyzed in this paper include:

1. *Sociodemographic characteristics:* age, sex, marital status, occupation (work status), education level, and household assets.
2. *Risk factors:* smoking, alcohol use, stressful events, sleep disorders, and body mass index (BMI).
3. *Self-reported chronic conditions:* self-reported diagnoses of chronic conditions (including angina, arthritis, cataract/eye sight problems, COPD, depression, diabetes mellitus, hypertension, and stroke).
4. *Objective measurements:* weight, height, visual acuity (using the Snellen charts), and blood pressure, measured three times in a sitting position.

Information from the interviews and assessments was used to describe health states that included diagnoses, risk factors, and impairments as described below. Disability was assessed using the 12-item version of WHODAS 2.0 questionnaire (35).

### Diagnoses

#### Hypertension

For all study participants, systolic and diastolic blood pressures were measured three times with participants in a sitting position using a Boso Medistar-S-wrist blood pressure monitor. An average blood pressure for the three readings was computed and used in the analysis. Hypertension was defined according to the World Health Organization (WHO) criteria (systolic blood pressure ≥140 mmHg and/or diastolic blood pressure ≥90 mmHg) (39).

For the conditions listed below, respondents were asked a range of questions on diagnosis and symptomatology for these chronic conditions, and their responses determined the diagnosis used here.

#### Diabetes mellitus, COPD, and eyesight problems/cataracts

For this analysis, prevalence estimates were based on the self-report of a doctor's diagnosis. Participants were asked the following questions: Have you ever been told by a doctor or a health worker that you have [condition]? If yes, were you started on treatment and are you still on treatment?

#### Stroke and angina

The prevalence for the conditions of stroke and angina was determined through algorithms using symptom-reporting (40, 41).

#### Depression

A diagnosis of depression was based on a diagnostic algorithm, with participant responses scored using the International Neuropsychiatric interview (MINI) criteria (42–44). The criteria used for determining depression were based on previous work using the MINI in Uganda (45, 46). The following screening questions for a major depressive episode were asked. For the past 2 weeks, were you depressed or down, most of the day, nearly every day? In the past 2 weeks, were you much less interested in most things or much less able to enjoy the things you used to enjoy, most of the time? If participants answered yes to these questions, they were asked a number of additional questions to ascertain a major depressive episode.

#### Arthritis

First, participants were asked if a health worker had ever diagnosed or told them that they have arthritis. If the answer was yes, they were asked about medication use or any other treatment for arthritis in the last 2 weeks and the last 12 months, and about symptoms, such as aching, stiffness, or swelling around the joints that were not related to injury and lasted for 1 month. Prevalence was determined using a diagnostic algorithm (40).

#### HIV

During WOPS1, participants were selected in the five categories described above. In order to avoid misclassification during WOPS2, all participants seen in WOPS1 who were previously HIV negative were subjected to repeat HIV testing. HIV testing was done using an algorithm for HIV-1 testing using three HIV-1 rapid tests as recommended by the Uganda Ministry of Health. The algorithm for HIV rapid testing consisted of an initial screening with the rapid test Determine HIV1/2. If the test result was negative the participant was given a diagnosis of HIV negative with no further rapid testing. If the test result was positive, the sample was retested with the rapid test HIV-1/2 Stat-Pak. If both tests gave a positive result the participant was given a diagnosis of HIV positive with no further rapid testing. If the tests gave discordant results (i.e. one positive and the other negative), the sample was further evaluated with the rapid test Uni-Gold Recombinant HIV-1/2. For those samples assessed by all three tests, two positive test results were interpreted as a positive diagnosis. If two of the three tests gave negative results, then the participant was diagnosed as being negative for HIV. The two resulting categories for our analysis below are those who tested HIV positive and those who tested HIV negative.

### Risk factors

Risk factors included tobacco use (if participants were using tobacco, they were asked about the duration of use), the method of tobacco consumption (whether they were smoking or using chew or snuff), and the quantity of tobacco consumed on each of the previous 7 days. Alcohol use was determined by asking whether participants had ever or were currently consuming alcohol, the duration of use, and the types of alcohol consumed. BMI was determined from weight and height measurements taken at the time of the survey. BMI was calculated by dividing weight in kilograms by height in meters squared.

### Disability

Questions necessary to generate the 12-item version of WHODAS 2.0 were asked in the interview (47–49). These questions gather information across six domains: cognition, mobility, self-care, getting along, life activities, and participation, asking about difficulty in these domains during the 30 days preceding the interview. The possible responses for each question were on a five-point scale: ‘none’, ‘mild’, ‘moderate’, ‘severe’, and ‘extreme or cannot do’. The WHODAS 2.0 algorithm was used to compute an overall score [range 0–100] for each respondent, with a higher score indicative of greater level of disability (47).

### Ethical issues

Ethical approval to conduct this study was obtained from the Uganda Virus Research Institute Science and Ethics Committee, the Uganda National Council for Science and Technology, and WHO's Ethical Review Committee. All participants gave a written and thumb-printed consent to participate in the study. For non-literate participants, an impartial third party *witnessed* the entire *consent* process and counter-signed the *consent* document on which the participant had placed their thumb-print.

### Statistical methods and data analysis

All analyses were conducted in Stata 13 (Stata Corp, College Park, Tx, USA). We did not use any imputation methods for missing data. However, the majority of variables had two or fewer missing cases, only three variables had more than 10 missing cases: BMI (11), stroke (12), and current employment status (17). All descriptive statistics and sample sizes are presented as un-weighted values, with a *p* value of <0.05 considered statistically significant (all *p* values are two-sided). We did not apply sampling weights. The study sample was selected randomly from lists of older people in the study population. Analyses for descriptive statistics and risk factors were stratified by HIV status for each of the following characteristics: sociodemographic variables (mean age, gender, locality, employment status, marital status, and highest level of education), all past and current use of tobacco, all past and current alcohol use, mean BMI, sleep problems, and antiretroviral (ART) use-conditional on HIV status. Analyses for chronic conditions (angina, arthritis, diabetes, COPD, depression, eye problems, hypertension, and stroke) were stratified by HIV status and age group; chi-square statistics highlight whether there were significant differences (1) across chronic conditions by HIV status and age group, and (2) significant differences between risk factors and HIV status. Median differences in age and BMI were calculated for the two respondent groups due to the data not being normally distributed. Wilcoxon rank-sum analyses were used to compare median differences in age and BMI for the two respondent groups. We conducted an ordered logistic regression and calculated predicted probabilities to show the differences in the number of chronic conditions across HIV status, gender, age group, and locality. We defined the number of chronic conditions using an algorithm that grouped respondents into three categories being zero chronic conditions; one condition; or two or more conditions. However, HIV was not considered a chronic condition for the purposes of these counts. We tested the proportional odds assumption for ordered logistic regression. This assumes that the coefficients that describe the relationship between the lowest versus all higher categories of the response variable are the same as those that describe the relationship between the next lowest category and all higher categories. For this, we used the omodel command in Stata and achieved a non-significant result, meaning that there was no difference in coefficients between models. For each respondent group, mean WHODAS 2.0 scores were determined for each chronic condition. T-tests were run within each respondent group to compare WHODAS scores for those with or without a chronic condition diagnosis.

Linear regression analyses were used to determine existing associations between sociodemographic factors, chronic conditions, and risk factors to WHODAS scores. Univariate analyses first determined significant main effects as well as interaction terms between HIV status and other factors before a multiple linear regression with these variables was undertaken. Although HIV was not significant in the univariate analysis, we left it in the final model as an a priori confounder together with age and gender. In the linear regression modeling, HIV negative was used as the reference category. Thus, compared to those who were HIV negative, HIV-positive individuals were expected to have higher WHODAS scores (meaning more disability). For all the univariate and multivariate analyses, a significance level of 0.05 was used. Model fit was assessed by examining residuals from the model. For this analysis, a robust regression analysis was used.

## Results

### Sociodemographic characteristics of study participants

Sociodemographic characteristics of the study population by HIV status are provided in Table 1. In total, the median age for the 471 participants was 63 (50–101). The majority of the sample was female (62.6%), widowed, still working, and had less than primary school education. About half of the study participants (51.8%) were HIV positive. The HIV-positive respondents tended to be younger. Only about 10% of older persons living with HIV were aged 70 or older, whereas over half of the HIV-negative sample was in the older age groups. Locality differences by HIV status are in part due to the sampling strategies.

**Table 1:** Sociodemographic factors by HIV status

	HIV+(*N*=244)		HIV–(*N*=227)	
		
Demographics	*N*	%	*N*	%
Gender				
Male	97	39.8	79	34.8
Female	147	60.3	148	65.2
Age				
50–59	135	55.3	33	14.5
60–69	82	33.6	69	30.4
70–79	23	9.4	82	36.1
80 +	4	1.6	43	18.9
Locality				
Wakiso	64	26.2	105	46.3
Kalungu	73	29.9	120	52.9
Masaka	107	43.9	2	0.9
Marital status				
Never married	3	1.2	9	4.0
Cohabitating/married	77	31.6	70	30.8
Divorced/separated	57	23.4	49	21.6
Widowed	107	43.9	99	43.6
Current employment status	(*n*=241)		(*n*=226)	
Still working	213	88.4	166	73.5
No longer working	28	11.6	60	26.6
Education level	(*n*=242)		(*n*=212)	
No formal education	35	14.5	53	23.5
Less than primary	96	39.7	113	50.0
Completed primary	43	17.8	16	7.1
Incomplete secondary	40	16.5	16	7.1
Completed secondary	15	6.2	14	6.2
Higher education than secondary	3	1.2	6	2.7
College/university or more	10	4.1	8	3.5

### Chronic conditions by HIV status

Several differences in the percentage of individuals reporting chronic conditions, other than HIV, were evident between the two respondent groups (Table 2). When comparing by HIV status, the prevalence of COPD and eye problems (except for those aged 60–69 years) were higher in the HIV-positive participants and prevalence of diabetes and angina were higher in HIV-negative participants. When comparing across age groups within HIV status, significant differences were present for eye problems and hypertension, which generally increased with age, and multi-morbidity for which the prevalence was higher in those with advanced age. The percentage of people with COPD decreased with age for both groups, with a higher starting point and a steeper decline in the percentage for the HIV-positive group.

**Table 2:** Percentage of chronic conditions by age and HIV status

	50–59 (*N*=168)		60–69 (*N*=151)		70+(*N*=152)			
					
	HIV+(*N*=135) (%)	HIV−(*N*=33) (%)	HIV+(*N*=82) (%)	HIV−(*N*=69) (%)	HIV+(*N*=27) (%)	HIV−(*N*=125) (%)	*p* Value by age	*p* Value by HIV status
Hypertension								
Yes	23.7	48.5	30.5	27.5	33.3	56.8	**0.00**	**0.00**
Diabetes								
Yes	2.2	9.1	0.0	8.7	3.7	8.1	0.287*	0.001*
Arthritis								
Yes	6.7	9.1	6.1	2.9	7.4	4.9	0.743*	0.316
Angina								
Yes	0.9	0.0	1.4	5.2	0.0	4.8	0.225*	0.05*
COPD								
Yes	10.4	3.0	7.3	1.5	3.7	1.6	0.026*	0.002*
Eye problems								
Yes	4.4	3.0	4.9	7.3	18.5	16.1	0.001*	**0.017**
Depression								
Yes	12.6	3.0	8.5	7.3	7.4	7.2	0.464	0.114
Stroke								
Yes	1.5	3.0	1.2	0.0	3.7	4.0	0.140*	0.533*
Number of conditions								
None	52.6	42.4	51.2	55.1	55.6	28.0	0.00*	0.004*
One	44.4	57.6	47.6	44.9	33.3	67.2		
More than one	3.0	0.0	1.2	0.0	11.1	4.8		

* Fisher's exact test used due to small cell size.

Note: HIV not treated as a chronic condition throughout all tables. Bold values are statistically significant at *p*<0.05.

The odds of having at least one or one or more, compared with no chronic conditions (other than HIV) are shown in Table 3. The odds of having one or more than one chronic condition were significantly higher in women and the oldest age group. The predicted probabilities of having one or more chronic conditions (other than HIV) in Table 4 give similar findings. Predicted probabilities are higher in women and in those aged 70 years and above.

**Table 3:** Ordered multivariate logistic regression of one or more chronic conditions^a^

Independent variable	OR (95% CI)	*p*
HIV status		
Positive*		
Negative	1.4 (0.9–2.2)	0.149
Gender		
Male*		
Female	1.6 (1.1–2.3)	*0.024*
Age group		
50–59*		
60–69	0.9 (0.5–1.4)	0.538
70 +	2.1 (1.2–3.6)	*0.006*
Locality		
Wakiso*		
Kalungu	0.5 (0.4–0.8)	*0.005*
Masaka	1.0 (0.6–1.8)	0.982

a Zero chronic conditions is the reference group. HIV status, gender, age group, and locality are included in the final model.

* values in italic are statistically significant at *p*<0.05.

**Table 4:** Predicted probabilities of one or more chronic conditions

	No chronic conditions	One chronic condition	More than one condition
Gender			
Male	0.52	0.46	0.01
Female	0.41	0.56	0.03
Age group			
50–59	0.50	0.48	0.02
60–69	0.54	0.44	0.02
70+	0.32	0.64	0.04
Locality			
Wakiso*	0.39	0.58	0.03
Kalungu	0.54	0.44	0.02
Masaka	0.39	0.58	0.03

* values are statistically significant at *p*<0.05.

### Risk factors by HIV status

Several significant differences in the percentage of respondents reporting or having risk factors for chronic conditions (other than HIV) by HIV status were also evident (Table 5). BMI was higher for HIV-negative respondents compared to those who were HIV positive. This, however, may be a result of HIV status rather than a risk factor for chronic conditions. A higher proportion of HIV-negative respondents said they currently use both tobacco and alcohol compared to HIV-positive respondents. A higher proportion of HIV-negative respondents also experienced mild sleep problems as compared to HIV-positive respondents.

**Table 5:** Risk factors by HIV status

	HIV+(*N=*244)		HIV−(*N*=227)		
			
	*N*	%	*N*	%	*p*
Ever used tobacco					
Yes	75	30.7	77	33.9	0.460
No	169	69.3	150	66.1	
Current user of tobacco (of ever users)					
Yes	16	21.3	37	48.1	<0.001
No	59	78.7	40	51.9	
Ever consumed alcohol					
Yes	198	81.2	171	75.3	0.126
No	46	18.9	56	24.7	
Currently consume alcohol (of ever users)					
Yes	59	29.8	74	43.3	0.007
No	139	70.2	97	56.7	
Sleep problems					
None	141	57.8	101	44.5	0.005
Mild	16	6.6	36	15.9	
Moderate	45	18.4	40	17.6	
Severe	28	11.5	35	15.4	
Extreme	14	5.7	15	6.6	
On ART	(*n*=212)				
Yes	192	90.6			
No	20	9.4			
				z score	
Median age	57		71	−11.5	<0.001
Median BMI	21.4		22.7	−4.0	0.001

### Linear regression of WHODAS scores

We found no interaction effects between HIV and other factors before undertaking the multiple regression analysis. Tables 5 and 6 show that there are several significant differences in the proportion of chronic conditions (other than HIV) and risk factors between respondents living with and without HIV. These reached significance in the univariate analyses (Table 5), however, when controlling for all other variables, many of the associations between these variables and the WHODAS score were no longer significant. These included current tobacco use, HIV infection, and arthritis diagnosis.

**Table 6:** Multivariable linear regression of WHO disability scores

Independent variable	Coefficient (95% CI)	*p*
Arthritis diagnosis		
No*		
Yes	−1.2 (−9.9 to 7.6)	0.795
Depression diagnosis		
No*		
Yes	9.4 (1.2 to 17.7)	0.025
COPD diagnosis		
No*		
Yes	6.6 (−2.2 to 15.4)	0.139
BMI	0.3 (−1.1 to 0.8)	0.152
Age	0.98 (0.7 to 1.2)	<0.001
HIV status		
Negative*		
Positive	−6.3 (−15.8 to 3.1)	0.187
Gender		
Male*		
Female	14.5 (7.8 to 21.2)	<0.001
HIV status/hypertension diagnosis		
HIV − /no diagnosis	−1.6 (−8.0 to 4.7)	0.605
HIV + /no diagnosis	3.4 (−3.2 to 9.9)	0.314
HIV status/gender		
HIV + /female	−6.8 (−15.5 to 1.8)	0.120
Current alcohol consumption		
Yes*		
No	4.7 (0.2 to 9.3)	0.04
Sleep problems (last 30 days)		
None*		
Mild	14.2 (7.3 to 21.0)	<0.001
Moderate	16.9 (10.8 to 23.0)	<0.001
Severe	20.3 (13.9 to 26.7)	<0.001
Extreme/can't do	21.7 (11.4 to 31.9)	<0.001
Currently employed		
Yes*		
No	6.8 (−0.2 to 12.3)	0.06

* Reference category.

Table 6 shows the factors that were significantly associated with WHODAS. A diagnosis of depression was associated with a 9.4 point (95% CI 1.2–17.7) increase in the WHODAS score, meaning a significant increase in disability compared to respondents who were not diagnosed with depression. A 1-year increase in the age of the respondent was significantly associated with a 1.0 (95% CI 0.7–1.2) increase in WHODAS score. Gender was also a significant factor relating to WHODAS scores with women having higher scores (14.5; 95% CI 7.8–21.2). Several risk factors were also associated with disability. Having a sleep problem of any type was significantly associated with higher WHODAS scores, with the more severe the sleeping problem, the higher the score. Respondents who had not consumed any alcohol in the past 30 days had, on average,a 4.7 point higher WHODAS score than current drinkers.

## Discussion

This study examines HIV status and non-HIV chronic conditions in Ugandans aged 50 years and over. The prevalence of chronic conditions (other than HIV) was affected by both age and HIV infection. When comparisons were made by age group, there were significant differences in the prevalence of COPD, eye problems, hypertension, and multi-morbidity which increased with age. When comparing by HIV status, there were significant differences, as seen for age. In addition, angina and diabetes were more common in those who were HIV negative. Reported multi-morbidity of chronic conditions was higher among respondents living with HIV than those not living with HIV, even after excluding HIV as a chronic condition.

Within African settings, there have been only three cohort studies (one in Uganda and two in South Africa) that have included a sufficient sample of HIV-positive individuals in order to assess the health and wellbeing of older people by HIV status (26, 50, 51). There are few studies from sub-Saharan Africa with which to compare our study findings. However, the pattern of a higher percentage of people with chronic conditions in HIV-negative older adults and in older age groups (70 years and more) was also observed in the WOPS1 data in both Uganda and in a comparable study from South Africa (50). In data from both these countries, the lower prevalence of hypertension in HIV-positive older adults was particularly striking (26). Hypertension was objectively measured through measurement of blood pressure. It is not very clear as to why HIV-negative older people have a higher prevalence of hypertension compared to their HIV-positive counterparts. It is possible that if HIV-positive older people are accessing more regular and better care, they may be more likely to have been told that they have another chronic condition, compared with HIV-negative older persons who may not be accessing health care as regularly. A study conducted among older people ‘infected or affected by HIV’ established that 90% of the HIV-positive older people were accessing treatment (52). The data available from WOPS2 on health care utilization show that 50% of the older people who were HIV negative had taken more than 1 year without visiting a health center. In future WOPS surveys, it will be important to complement self-reported diagnoses of chronic diseases/impairments with objective measures to see whether these differences persist. Furthermore, the age differences between the two groups (HIV positive and HIV negative) might also be driving the differences in chronic conditions. Those who were HIV positive were younger compared to those who were HIV negative, with only a small proportion of the HIV-positive respondents (11%) aged 70 years and over. However as Table 2 illustrates, while older age is associated with the reporting of chronic conditions generally, for some chronic conditions, HIV infection is also an important factor.

Tobacco use and alcohol consumption did not differ between HIV-positive and HIV-negative older people (53). A study conducted in rural areas of three African countries showed that alcohol consumption and tobacco smoking were significantly higher in men and women aged 50 years and over than in those under age 50; however, that study did not collect information on respondents’ HIV status (11). While health behaviors and individual factors increase the risk of chronic conditions, it is also important to note that a majority of older persons in low-income countries are poor and have access to limited health resources. For example, poor living conditions are a major risk factor for chronic respiratory diseases (54, 55). Further, in many LMICs, due to poverty and mobility issues, older persons are unable to seek medical attention for the early detection and treatment of these chronic conditions even though they recognize symptoms or understand that the condition is treatable (56). Further, the quality and quantity of services related to chronic conditions, particularly related to the needs of older persons, are limited in the majority of LMICs (21). Thus, it is important to highlight that the need for health service and structural changes as well as the lack of available services contribute significantly to the quality of life for those living with chronic conditions (21).

When we looked at disability using WHODAS2.0 scores, sleep problems and depression were significantly related to higher scores (higher reported disability). While it is not clear from our data if these are causing disability or if disability is causing these problems, sleep and mental health are arguably among the most under-reported illnesses in lower level health facilities, particularly for older adults. This calls for a greater focus on mental health, and investigations into why these issues exist among older Ugandans. Some of the reasons that have been previously cited for poor mental health among older Africans include lack of social connection, family support, HIV stigma, and caregiving burden (57, 58). There is also need to examine best practices to treat mental health issues in older people at lower levels of the health care systems and through community-based interventions.

Confirming findings from WOPS1 (26), women had significantly higher disability scores than men. It is unclear why older women have these higher scores since there is evidence that adult women generally have better health seeking behavior than men (59–61). However, there is evidence that older African women report poorer self-rated health and quality of life than men, both of which are associated with disability (51, 62). This relationship could be related to various aspects of home and social life including older women's care giving responsibilities and the interrelationship between mental and physical health (63–65). The underlying reasons for older women having significantly higher disability scores than older men need further research. Respondents who reported not consuming alcohol in the past 30 days reported higher WHODAS score than current drinkers. Although the odds ratio for those who were currently not consuming alcohol was high, the confidence intervals were wide with the lower limit of 0.2. One possible explanation may be that those who already knew they had chronic conditions were abstaining from alcohol and tobacco use because of their chronic condition. Given the preponderance of evidence of the role of alcohol, tobacco, and diet in chronic conditions in high-income countries (66–68), it will be important to track these relationships over time.

### Strengths and weaknesses

This study has potential strengths and weaknesses. There are very few studies in Uganda and indeed sub-Saharan Africa that examine the differences in chronic conditions between older people living with and without HIV. This study provides initial data on chronic conditions, including the prevalence of the risk factors and the association between chronic conditions and disability, in older people living with and without HIV in Uganda.

One limitation of these data is that most of the diagnoses made were by self-report. Though these may not be as accurate as diagnoses made by clinicians, diagnoses by self-report have been widely used in other studies (2, 38, 40). It will be important to continue to explore and validate self-reports of various health conditions and behaviors against more objective measures in these and other data from sub-Saharan Africa. In addition, because of anticipated mortality and loss to follow-up in the original sample of WOPS1, we added 100 respondents who were HIV positive in the WOPS2 sample. These new respondents might be different in a number of ways from the original WOPS1 sample, and from other HIV-positive individuals living in Uganda, as they were identified through an NGO that serves people living with HIV. Last, there were age differences between the HIV-positive and negative groups with the HIV positive being younger than the HIV negative; however, to manage this in the regression models, we controlled for age.

## Conclusion

In conclusion, this study has identified a number of factors, like sleep problems and depression, and COPD among HIV-positive individuals, which are associated with high disability scores among older Ugandans. Unfortunately, in the majority of lower level health centers in Uganda, which are the first levels of care for most of the older people, such factors are under-reported, and there are not adequate resources for services to address these problems. As the population of Uganda ages, with and without HIV, there is need to revise Ugandan health policy to consider the health needs of older people. It is essential to begin focusing on community and health service interventions that positively impact both physical and mental health in order to reduce disability and improve overall quality of life among older Ugandans.

## Paper Context

Previous work on this topic has focused on chronic conditions in HIV-negative older people. This paper adds new information on chronic conditions and their impact on disability in HIV-positive and HIV-negative older people. We recommend that health care workers should always look for symptoms and signs of chronic disease in older people irrespective of their HIV status.
